# Paraventricular Hypothalamic Mechanisms of Chronic Stress Adaptation

**DOI:** 10.3389/fendo.2016.00137

**Published:** 2016-10-31

**Authors:** James P. Herman, Jeffrey G. Tasker

**Affiliations:** ^1^Department of Psychiatry and Behavioral Neuroscience, University of Cincinnati, Cincinnati, OH, USA; ^2^Department of Cell and Molecular Biology, Tulane Brain Institute, Tulane University, New Orleans, LA, USA

**Keywords:** corticotropin-releasing hormone, vasopressin, hypothalamo–pituitary–adrenal axis, glucocorticoids, neuroendocrine system

## Abstract

The hypothalamic paraventricular nucleus (PVN) is the primary driver of hypothalamo–pituitary–adrenocortical (HPA) responses. At least part of the role of the PVN is managing the demands of chronic stress exposure. With repeated exposure to stress, hypophysiotrophic corticotropin-releasing hormone (CRH) neurons of the PVN display a remarkable cellular, synaptic, and connectional plasticity that serves to maximize the ability of the HPA axis to maintain response vigor and flexibility. At the cellular level, chronic stress enhances the production of CRH and its co-secretagogue arginine vasopressin and rearranges neurotransmitter receptor expression so as to maximize cellular excitability. There is also evidence to suggest that efficacy of local glucocorticoid feedback is reduced following chronic stress. At the level of the synapse, chronic stress enhances cellular excitability and reduces inhibitory tone. Finally, chronic stress causes a structural enhancement of excitatory innervation, increasing the density of glutamate and noradrenergic/adrenergic terminals on CRH neuronal cell somata and dendrites. Together, these neuroplastic changes favor the ability of the HPA axis to retain responsiveness even under conditions of considerable adversity. Thus, chronic stress appears able to drive PVN neurons *via* a number of convergent mechanisms, processes that may play a major role in HPA axis dysfunction seen in variety of stress-linked disease states.

## Introduction

The hypothalamo–pituitary–adrenocortical (HPA) axis is required for appropriate adaptation to external or internal challenge. The so-called HPA axis “stress response” culminates in the release of glucocorticoids by the adrenal gland, which acts at multiple sites throughout the body (and brain) to mobilize energy resources. This redistribution of energy provides essential fuels for meeting real or anticipated challenges to homeostasis or well being (i.e., “stressors”) (1). While important for immediate adaptation, prolonged exposure to glucocorticoids can create a metabolic challenge of its own. Consequently, activation of the HPA axis is kept in control by negative feedback, wherein glucocorticoids inhibit their own release (2).

**Figure 1:**
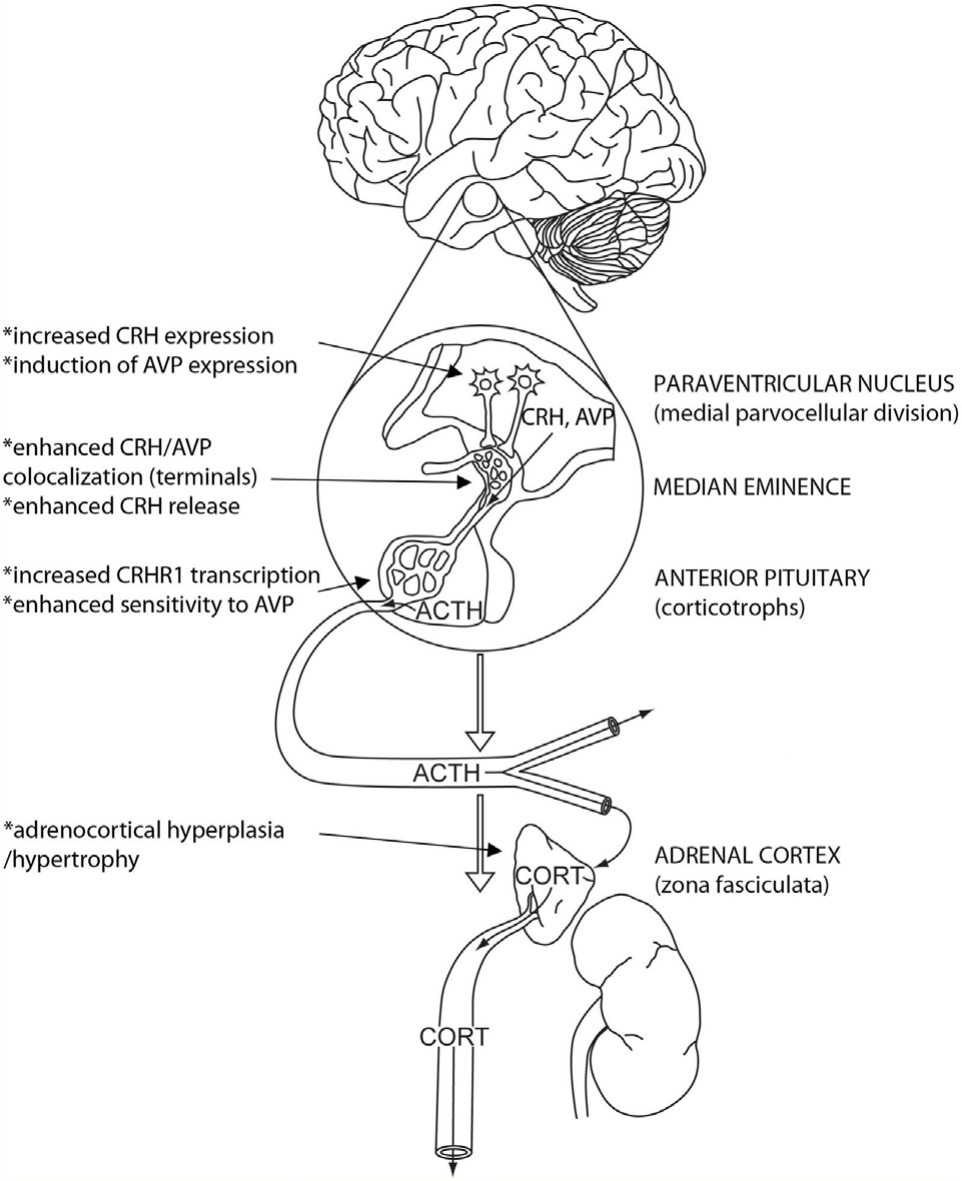
**Plasticity across the hypothalamo–pituitary–adrenocortical (HPA) axis following chronic stress**. HPA axis activation is initiated by CRH (and AVP)-containing neurons in the medial parvocellular paraventricular nucleus (PVN), which releases ACTH secretagogues into hypophysial portal vessels in the median eminence. ACTH is released from the pituitary and travels *via* the systemic circulation to the adrenal cortex, where it causes the synthesis and secretion of glucocorticoids [e.g., cortisol or corticosterone (CORT)]. Under conditions of chronic stress, there are marked changes in cellular reactivity across the HPA axis, including (1) increased CRH and AVP expression in, and enhanced excitability of CRH neurons in the PVN, (2) increased CRH/AVP colocalization and CRH release in the median eminence, (3) increased CRHR1 and V1b gene expression in and sensitivity to CRH and AVP of pituitary corticotropes, and (4) adrenal hypertrophy and enhanced adrenal sensitivity to ACTH. All changes are consistent with increased potential for HPA axis activation. Modified from Myers et al. (4), with permission.

The HPA axis is controlled by peptidergic neuroendocrine neurons located in the medial parvocellular division of the hypothalamic paraventricular nucleus (PVN) (Figure 1). These neurons are responsible for the release of corticotropin-releasing hormone (CRH), which acts as the obligate factor responsible for adrenocorticotrophic hormone (ACTH) release in most organisms (3). Upon stimulation, CRH is released into hypophysial portal vessels in the external lamina of the median eminence, where it is transported to the anterior pituitary gland to promote ACTH release. ACTH then travels *via* the systemic circulation to the adrenal cortex, where it triggers the synthesis and release of glucocorticoids.

Importantly, PVN CRH neurons also synthesize arginine vasopressin (AVP), which is co-stored and co-released with CRH in the median eminence (5, 6). While having minimal effects on its own, AVP can synergize with CRH to greatly amplify ACTH release (7). Under unstimulated conditions, AVP expression in CRH neurons is very low and likely plays a minimal role in ACTH secretion. However, following prolonged activation (e.g., adrenalectomy or as we will see below, chronic stress) (8, 9), parvocellular AVP production is markedly increased, suggesting a role in chronic drive of the HPA axis. Electron microscopy studies indicate depletion of AVP from CRH terminals in the median eminence following acute stress (10), consistent with co-release in response to drive of PVN neurons.

Both CRH and AVP act at the level of the pituitary corticotrope to modulate the release of ACTH. CRH binds to corticotropin-releasing hormone R1 receptors (CRHR1), causing activation of adenylate cyclase and subsequent release of ACTH (11). Deletion of the CRH gene blocks both basal and stress-induced ACTH release, indicative of the obligatory nature of CRH for HPA axis activation (12). In contrast, AVP does not drive ACTH release on its own but complements the actions of CRH (*via* binding to AVP1B receptors) (13). Together, CRH and AVP provide for a broad range of corticotrope response following PVN stimulation.

It is important to note that a subset of PVN CRH neuron project centrally and may be of functional importance in behavioral regulation. Lesions of the parvocellular PVN reduce anxiety-like behaviors in a novel environment, suggesting a role in emotional regulation (14). A recent study indicates that optogenetic inhibition of PVN CRH neurons reduces stress-induced grooming and enhances locomotion and rearing following stress, whereas stimulation induces grooming and reduces exploratory behaviors (15). Collectively, these data suggest that PVN CRH (and possibly AVP) neurons may be involved in coordinating behavioral as well as neuroendocrine responses to stress.

Parvocellular CRH neurons also express numerous other neuropeptides, including angiotensin II, cholecystokinin, and neurotensin (16). The role of these other peptides in HPA axis function has yet to be clarified. In addition, parvocellular PVN neurons have the capacity to release the excitatory neurotransmitter glutamate (17). Given the presence of presynaptic glutamate receptors in the median eminence, it is possible that glutamate may also influence local release of peptide at the level of the neurovascular junction (18). Indeed, blockade of GluR5-containing kainate receptors in the median eminence inhibits stress-induced ACTH release (19), suggesting a role for local glutamatergic signaling in HPA axis control.

The PVN is one of the primary sites of glucocorticoid negative feedback regulation of the HPA axis. Negative feedback is largely mediated by glucocorticoid receptors (GRs), which are activated mainly when glucocorticoid levels are elevated (e.g., during stress responses) (20). The GR is richly expressed in the medial parvocellular PVN and is co-localized with CRH (21, 22), placing it in prime position to control output of the very neurons that activate the HPA axis. Within the PVN, GR-mediated negative feedback of CRH neuronal activation (“fast feedback”) is likely mediated by non-genomic glucocorticoid signaling at or near the cell membrane (23, 24). Rapid inhibition of CRH neurons is key to limiting the duration of glucocorticoid secretion following acute stress, as genomic feedback would not be sufficiently fast to terminate HPA axis activation in a timely fashion. Fast feedback is mediated by glucocorticoid-dependent mobilization of endocannabinoid production in putative CRH neurons, which cause inhibition of presynaptic glutamate release *via* retrograde signaling at type 1 cannabinoid (CB1) receptors (23). While there is evidence to suggest that membrane effects involve the classical GR (25, 26), the exact mechanism of glucocorticoid action remains to be delineated (27).

Genomic feedback effects on CRH neurons likely occur at longer poststimulation latencies and may be mediated by ligand-dependent nuclear translocation and subsequent interactions of the GR with cognate DNA binding elements or other transcription factor complexes. Genomic actions of GR may be involved in glucocorticoid-mediated inhibition of CRH and AVP gene expression in hypophysiotrophic neurons following adrenalectomy (28, 29). However, there are data to suggest that rapid effects of stress on CRH gene transcription may be glucocorticoid-independent. Rapid inhibition of CRH heteronuclear RNA (hnRNA) expression appears to be mediated by rapid stress-induced increases in expression of the inducible cyclic AMP early repressor (ICER) isoform of the cyclic AMP response element modulator (CREM), which binds to the CRH promoter and blocks transcription (30). Transcriptional repression of CRH by ICER/CREM is not dependent on a glucocorticoid surge, suggesting an alternative mechanism.

Glucocorticoids also signal through the mineralocorticoid receptor (MR). The MR is occupied at low circulating levels of glucocorticoids and is not thought to mediate glucocorticoid feedback effects (20). However, there are data suggesting that occupation of the MR is required for appropriate regulation of stress responses in some contexts (31), and certain rapid glucocorticoid actions in the hippocampus and basolateral amygdala are mediated by MR activation (32, 33). While there is evidence for MR expression in the medial parvocellular PVN (34), its role in the local regulation of HPA axis function is unexplored.

## Chronic Stress-Induced Neuropeptidergic Plasticity in the PVN

Chronic exposure to stress produces regimen-dependent alterations in PVN production of CRH mRNA. Most stressors that involve non-social interventions (e.g., repeated immobilization, repeated footshock, chronic unpredictable/variable stress, repeated predator exposure) produce upregulation of PVN CRH mRNA expression (9, 35–37). For example, in our hands, we have observed increased PVN CRH mRNA expression in all studies using chronic variable stress (1- to 4-week exposure to a random assort of stressors twice/day, at unpredictable times) (38–41). Indeed, the magnitude of CRH mRNA upregulation is remarkably similar across studies (generally falling between 40 and 60%), despite studies being conducted at two different institutions and over several years (38–41). Thus, it is evident that increased drive to the PVN effectively enhances CRH gene expression in these regimens. The exact mechanism driving elevations in CRH gene expression is not completely understood but probably involves increases in transcription mediated by repeated activation of cAMP (42, 43). Transcription may be partially (but not completely) counter-balanced by enhanced glucocorticoid-mediated degradation of CRH mRNA (44), likely as a feedback homeostatic control mechanism.

The impact of chronic stress on CRH gene transcription is accompanied by enhanced CRH peptide production in PVN cell bodies and decreased storage in the median eminence (45). The latter observation suggests an increase in turnover of CRH, which is consistent with chronic activation. In the cell soma, increased peptide synthesis is accompanied by an increase in the physical size of the CRH neurons (46), similar to that seen in magnocellular AVP and oxytocin neurons following dehydration and lactation (47, 48). Increased cell size may reflect a general drive on transcription and protein synthesis, perhaps as a way of adapting to increased energetic demand. These data are consistent with increase in protein synthesis as well as in capacity for release, which may partially underlie baseline glucocorticoid hypersecretion and stress facilitation seen following chronic stress (below).

Chronic stress also increases AVP gene transcription in parvocellular PVN neurons (49). The dynamics of chronic stress-induced AVP and CRH transcriptional responses differ considerably. Exposure to habituating, homotypic stressors (e.g., restraint) results in the loss of PVN CRH transcriptional responses over time, in terms of both hnRNA and mRNA expression (50). Moreover, CRH neurons lose the ability to mount transcriptional responses to a novel acute (heterotypic) stress (e.g., hypertonic saline) following repeated stress exposure. In contrast, while AVP hnRNA and mRNA responses also habituate with repeated restraint, robust transcriptional responses are observed when challenged with a heterotypic stressor, stronger than that seen in stress-naïve controls (49). Together, the data suggest that AVP may play an important role in maintaining or potentiating PVN (and by extension, HPA axis) drive after homotypic stress exposure.

Chronic variable stress reduces expression of the GR in the medial parvocellular PVN. Since both CRH and AVP gene/protein expression are negatively regulated by glucocorticoids, it is possible that reduced GR may play a role in the observed upregulation of both genes during chronic stress. Indeed, expression of GR is negatively correlated with CRH mRNA in the PVN (9, 51), suggestive of a mechanism for stress-induced upregulation. However, it is important to note that downregulation of GR mRNA is not observed in all stress regimens, despite elevated CRH, suggesting that other mechanisms may also contribute to driving PVN gene expression. In this regard, it is important to note that the efficacy of GR signaling may be negatively modulated by posttranscriptional mechanisms. For example, nuclear GR signaling can be affected at the level of receptor translocation (as seen in aging) (52) or binding to nuclear factors that modulate transcriptional activity (53). Regarding the latter possibility, GR binding to different isoforms of steroid receptor coactivator protein 1 can dramatically affect the transcription of CRH and CRH gene methylation (54).

## Cellular Impact of Chronic Stress on Medial Parvocellular PVN Neurons

**Figure 2:**
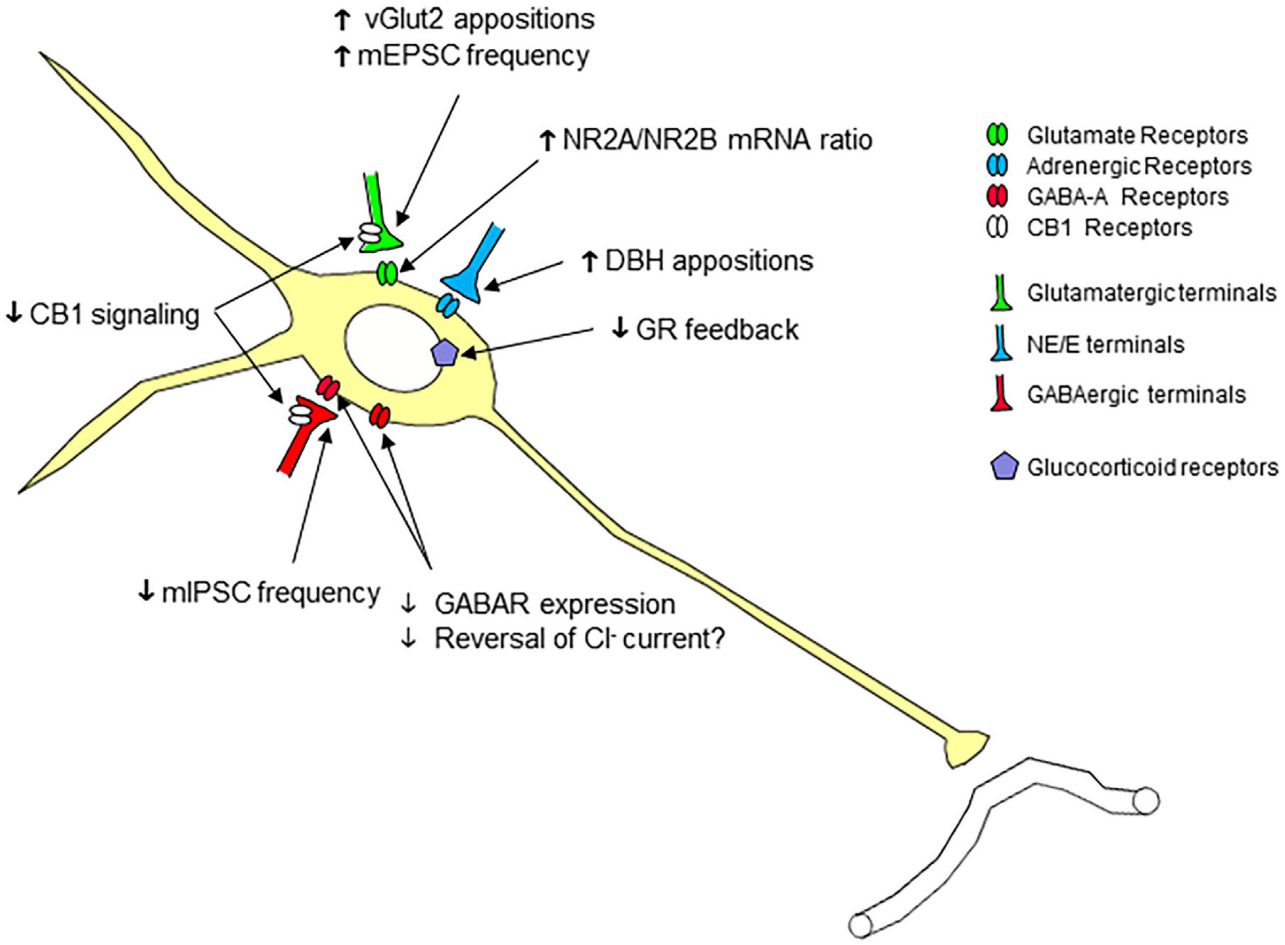
**Cellular plasticity in parvocellular paraventricular nucleus neurons following chronic stress**. Chronic stress causes a number of neuroplastic changes in parvocellular neurons consistent with increased excitability, including (1) increased number of (excitatory) glutamergic (VGluT2) terminal appositions, (2) enhanced excitatory synaptic inputs (increase in mEPSC frequency), (3) enhanced NR2A/NR2B ratio, predictive of increased NMDA receptor calcium permeability, and (4) increased number of (putative excitatory) norepinephrine/epinephrine terminal appositions. In addition, there is evidence for reduced inhibition of the PVN neurons, in terms of (1) reduced GABAergic synaptic inputs (decrease in mIPSC frequency), (2) decreased expression of synaptic and extrasynaptic GABA_A_ receptor subunits, (3) decreased cannabinoid type 1 receptor signaling, and (4) reduced GR expression/signaling, in some, but not all, studies.

Chronic stress produces multiple cellular adaptations in CRH neurons that are consistent with enhanced excitability (Figure 2). Chronic variable stress reduces mIPSC frequency (but not amplitude) in putative CRH neurons, indicative of loss of inhibitory inputs (55, 56). Chronic variable stress exposure also decreases the expression of GABA_A_ receptor α5, β1, β2, and δ subunits (55, 57), consistent with reduced GABAergic inhibition. Importantly, receptors expressing δ subunits are largely extrasynaptic and are thought to mediate tonic inhibition (58). The loss of extrasynaptic receptors, therefore, would be expected to reduce tonic GABAergic inhibition of PVN neurons. In addition, there is evidence supporting a reversal of chloride current in CRH neurons after stress exposure, essentially rendering GABA less effective at the GABA_A_ receptor and resulting in reduced synaptic inhibition (59). Loss of inhibitory GABAergic transmission is linked to marked glucocorticoid-dependent downregulation of the potassium-chloride co-transporter, KCC2, resulting in enhanced intracellular chloride ion concentration and a weakened membrane chloride gradient (59). Notably, chronic variable stress also causes downregulation of KCC2 in CRH neurons, consistent with lasting impairments in inhibitory GABA signaling (60).

There is evidence to suggest enhanced excitability of PVN neurons following chronic stress. Baseline excitatory drive of parvocellular PVN neurons is enhanced by chronic stress exposure, manifest as increased mEPSC frequency (56). In addition, ionotrophic *N*-methyl-d-aspartate receptor subunits are modulated by chronic variable stress in a manner consistent with enhanced postsynaptic glutamate receptivity. Chronic stress causes a selective decrease in expression of NR2B in the medial parvocellular PVN, whereas NR1 and NR2A expression are unaffected. As calcium permeability of NR2A containing channels is greater than that of NR2b channels, these data predict an enhanced excitability of the NMDA receptor complex following chronic stress (61).

Enhanced PVN excitability may also be affected by chronic stress-induced loss of endocannabinoid signaling in PVN afferents. Animals exposed to chronic restraint stress lose depolarization-induced suppression of both glutamate and GABA release at parvocellular PVN neurons (62). Given the reversal of the chloride gradient following chronic stress, both effects are consistent with reduced inhibition of PVN neurons. Loss of inhibitory control appears to be mediated by a GR-dependent inhibition of CB1 receptor signaling, likely mediated by the loss of CB1 terminals on PVN neurons. Thus, chronic restraint stress likely produces a loss of presynaptic cannabinoid inhibition, effectively blocking endocannabinoid-mediated glucocorticoid-dependent fast feedback inhibition of the HPA axis (62). However, it is important to note that glucocorticoid-induced endocannabinoid signaling is not affected following chronic variable stress (56), indicating that different mechanisms may regulate PVN responses under conditions of predictable vs. unpredictable stress exposure.

Chronic stress exposure affects markers of cellular activation in the PVN. Neuronal activation causes rapid induction of the immediate early gene, *cfos*, largely as the result of depolarization (63, 64). The resulting protein, Fos, binds with other cofactors of the *jun* family to form a potent transcriptional activation complex acting at activator protein 1 (AP1) response elements on DNA (65). Not surprisingly, acute stress produces a marked increase in PVN *cfos* mRNA/Fos expression, consistent with cellular activation (63, 64). However, the acute Fos response to homotypic stressor exposure (e.g., restraint, noise) wanes over repeated exposure (66–68), suggesting habituation of cellular activation. In contrast, PVN *cfos* gene expression/Fos protein induction by a heterotypic stressor is preserved or enhanced in some paradigms [e.g., chronic cold plus acute restraint (69)] but not others [e.g., chronic variable stress plus novelty, chronic social stress plus open arm (elevated plus maze) exposure] (70, 71). The fact that the acute Fos activation is reduced in the latter paradigms indicates some degree of cross-habituation to stressor exposure, at least as far as Fos induction is concerned. The lower *cfos* mRNA/Fos response may also be associated with the relative (low) intensity of the evocative stimuli with respect to other stimuli occuring in the unpredictable stress or social stress paradigms. Of course, it is important to consider that the magnitude of the Fos response may not be an accurate reporter of cellular activation in the context of repeated drive, given that other intracellular changes [e.g., increased CRH expression (68), enhanced excitability (56)] may compensate for habituation in the AP1 pathway.

Chronic stress also causes enduring changes in immediate early gene family members, consistent with chronic activation. For example, expression of FosB/delta FosB, a Fos family member that aggregates in the cell over repeated activations, is upregulated in the PVN by morphine withdrawal (72).

## Chronic Stress Neuroplasticity of PVN Inputs

Chronic stress-induced enhancement of PVN response capacity may be driven in large part by neuroplasticity in afferent connections (Figure 2). Work from our group suggests that chronic variable stress increases glutamatergic inputs to CRH neurons, as determined by elevations in the number of vesicular glutamate transporter 2 (VGluT2) appositions onto both dendrites and somata (46) and increases in excitatory synaptic inputs to PVN neurons (56). Both observations suggest that the enhanced drive is mediated by additional synaptic contacts. Moreover, early-life maternal enrichment, which is linked to reduced stress reactivity, decreases VGluT2 synaptic contacts and excitatory synaptic inputs in CRH neurons (73), further suggesting that the glutamate innervation is an important determinant of PVN excitability.

Chronic stress also increases the number of dopamine beta hydroxylase-positive appositions with CRH dendrites and somata, consistent with enhanced noradrenergic (and adrenergic) innervation (46). A sizable body of evidence suggests that PVN norepinephrine (NE) drives HPA axis responses in a stressor-dependent fashion (74, 75), suggesting the capacity for stress-induced enhancement of NE innervation to drive CRH neurons. Lesion of PVN-projecting NE/E neurons using DBH-conjugated saporin (DSAP) reduces ACTH responses to an acute stress in rats previously exposed to chronic variable stress, indicating that adrenergic input is required for the excitatory drive of CRH neurons under these conditions. However, corticosterone responses are not affected by PVN NE/E depletion, consistent with compensation at the level of the adrenal gland (see below) (76). Notably, DSAP also blocks chronic stress-induced increases in PVN synaptophysin and VGluT2 expression, suggesting that lesions may interfere with glutamatergic neuroplasticity (76).

Subsequent studies have compared the impact of chronic stress on PVN innervation in males and females. Overall, medial parvocellular PVN innervation, determined from the density of synaptophysin staining, is greater in females than in males. However, in response to chronic stress, synaptic density markedly increases in males but decreases in females (77). Enhanced PVN innervation in males is accompanied by increased VGluT2 innervation of CRH neurons (unpublished data). Despite clear evidence for chronic stress-induced decrements in inhibitory synaptic inputs (55, 56), we did not observe a loss of GABAergic appositions onto CRH neurons in either males or females (unpublished data). These results suggest that the loss of GABAergic input to PVN neurons may not be simply due to reduced synaptic afferents but rather may reflect differences in release probability. Chronic stress effects on GABAergic inputs may also be connected to modifications in GABAergic signaling efficacy, mediated by sex differences in postsynaptic reversal of chloride gradients or reduced efficacy of presynaptic CB1 signaling, noted above. These data imply a marked difference in PVN synaptic physiology in males and females, the significance of which remains to be evaluated.

Not all presynaptic changes are linked to enhanced excitability. Work from our group has demonstrated that glucagon-like peptide 1 (GLP-1) inputs to the PVN (from the caudal nucleus of the solitary tract) are powerful drivers of HPA axis stress responses (78, 79). Moreover, exogenous GLP-1 enhances HPA axis facilitation following chronic variable stress, suggesting a role in driving CRH neuronal responsiveness (79). However, chronic stress results in profound inhibition of the expression of the GLP-1 precursor preproglucagon (PPG) in NTS neurons, accompanied by loss of GLP-1 innervation to the medial parvocellular PVN (80). Reduction of PPG gene expression is glucocorticoid-dependent, suggesting that chronic stress-induced glucocorticoid secretion removes an important excitatory drive to the PVN (80). Removal of GLP-1 PVN innervation may represent a feedback mechanism that limits excessive drive of the HPA axis following chronic stress.

## Chronic Stress and PVN Afferents

The PVN gates output of the HPA axis, acting essentially as a “final common pathway.” Consequently, PVN output is subject to upstream changes in stress-sensitive projections (81). Lesion studies suggest that damage to key limbic structures following chronic stress causes marked changes in PVN activation. Damage to the posterior subregion of the bed nucleus of the stria terminals potentiates HPA axis stress responses and increases PVN *cfos* gene expression following chronic variable stress. The posterior BST provides strong GABAergic input to the PVN, suggesting that chronic stress reduces BST inhibition of the HPA axis (82). However, lesions of the anterior subregion of the BST reduce HPA axis responses to acute stress but enhance ACTH release and PVN *cfos* mRNA expression following chronic stress, consistent with a change in the functional connectivity of this region with the PVN as a result of repeated stress (83). The anterior BST sends both excitatory (CRH-containing) and inhibitory (GABAergic) projections to the PVN (84, 85), raising the possibility that the relative weighting of these inputs is modified by repeated stress exposure.

Lesions of the paraventricular thalamic nucleus (PVT) have profound effects on both habituation and sensitization of the HPA axis and PVN responses to stress (86). This region of the limbic thalamus does not connect directly with the PVN and thus requires intermediary relays to access CRH neurons. Damage to the PVT has no effect on acute stress responses but blocks chronic stress-induced facilitation of HPA axis responses and PVN Fos induction to a novel stress (86). Moreover, damage to this region blocks habituation of HPA axis responses to repeated stressor exposure (86). These data indicate a role for this structure in relaying information on stress chronicity to the PVN.

It is of interest to note that regions controlling acute stress reactivity do not necessarily modulate chronic stress responses. Lesions of the central nucleus of the amygdala, locus coeruleus, or ventral subiculum have no effect on somatic end points, HPA axis activity, or PVN neuropeptide plasticity following chronic stress, despite profound actions on acute stress reactivity (38, 39). Thus, circuits mediating PVN plasticity following chronic stress may differ from those controlling acute reactivity.

We recently used FosB immunostaining to map brain regions selectively activated by chronic variable stress, as compared to a chronic heterotypic (habituation) stress regimen (restraint). Our data identified a small set of interconnected structures that expressed FosB after chronic variable stress: the infralimbic cortex (IL), posterior hypothalamus, and NTS (87). Both the posterior hypothalamus and NTS are connected with the IL and send excitatory projections to the PVN (88–90), consistent with a possible role in potentiation of the excitatory drive to the HPA axis following chronic stress.

Selective inhibition of GR signaling further implicates two of these brain regions, the IL and NTS, in alterations to PVN drive by chronic stress. Lentiviral knockdown of GR in the IL (but not prelimbic cortex) produces a marked potentiation of HPA axis and PVN Fos induction to a novel stress in animals exposed to CVS (91), a phenomenon linked to interneuron-mediated decreases in IL output (92). Implants of the GR antagonist mifepristone in the NTS inhibit PVN excitability following chronic stress, suggesting that the glucocorticoids may play a role in inhibiting the NTS drive to the PVN (93). These data implicate both regions as sites of glucocorticoid feedback (perhaps at the genomic level) and suggest that they function to set HPA reactivity consequent to chronic stress.

## Impact of Chronic Stress on PVN Output

Chronic stress leaves a cumulative record of HPA activation, including adrenal hypertrophy (due to elevated ACTH exposure), thymic atrophy (as a result of glucocorticoid-mediated cell death), and increased PVN FosB/delta FosB expression. All effects are believed to be a direct consequence of central drive of CRH neurons by stressors. However, it is important to consider the dynamics that underlie the progression of chronic stress endpoints. In general, the largest somatic and HPA axis effects are observed in the first few days of a chronic stress regimen. For example, exposure to chronic social stress causes a profound initial weight loss that is then maintained across the duration of the stress regimen (94). Similarly, increases in resting glucocorticoid secretion are greatest across the first few days of chronic stress and can diminish significantly over time. Given evidence for adaptation within chronic stress regimens, it is likely that the enduring effects of chronic stress on PVN activation are mediated by a combination of marginal increases in baseline drive and episodic activation of CRH neurons as a result of each stress exposure.

It is important to note that chronic stress activation of the HPA axis may be adjusted downstream of the CRH neuron (Figure 1). Chronic stress results in marked changes in pituitary corticotropes, including an upregulation of proopiomelanocortin (ACTH precursor protein) synthesis and increases in both CRHR1 and vasopressin 1b (V1b) receptor mRNA expression (13, 95), suggesting mechanisms that would enhance capacity for ACTH release. Interestingly, chronic stress triggers increases in pituitary V1b, but not CRHR1 binding, again predicting a role for AVP in potentiation of stress responses at the level of the pituitary (11). We recently reported an increase in the sensitivity of pituitary ACTH secretion to CRH stimulation *in vivo* and *in vitro* following chronic variable stress (56).

Chronic stress also causes changes at the level of the adrenal cortex. Exposure to 2 weeks of chronic variable stress results in marked hyperplasia and hypertrophy of the adrenal gland, as well as a marked enhancement of adrenal sensitivity to ACTH (96). Prior studies indicated that adrenal sensitivity to ACTH is modified by splanchnic nerve cuts (which remove innervation of the adrenal cortex) (97), suggesting that chronic stress-related changes in adrenal size and responsiveness are mediated by increased sympathetic activation.

There are marked differences in HPA axis regulatory mechanisms in males and females. Basal total corticosterone levels are higher in females than in males [e.g., Figueiredo et al. (98)]. However, circulating corticosteroid-binding globulin is higher in females than males, with the net effect of negating the sex differences in resting corticosterone levels (however, stress-induced free corticosterone remains higher in females than males) (99). In addition, estrogens play a major role in driving HPA axis activation in females and are responsible for elevated glucocorticoids during proestrus and estrus in rodents (98, 100, 101). Interestingly, the effect of estrogens on the HPA axis is particularly pronounced at the level of the adrenal gland: estradiol inhibits restraint-induced ACTH release but strongly enhances the sensitivity of the adrenal to ACTH, which results in a net increase in stress-induced glucocorticoid release (102).

## Alternative PVN Responses to Chronic Stress: Social Subordination

While upregulation of PVN function is commonly observed after most chronic stress regimens, it is important to note that stress regimens with strong social or metabolic components produce more variable results. For example, in rats, chronic social stress appears to decrease PVN CRH mRNA expression in subpopulations of subordinate individuals (103). Decreased CRH mRNA is observed in individuals that exhibit short defeat latencies to repeated social defeat and in individuals showing a stress-non-responsive phenotype in a colony-based subordination paradigm (104), suggesting an enhanced downregulation of response capacity in the most severely affected animals. In mice, results have been more variable. Some studies report upregulation of CRH mRNA, particularly in subsets of animals that do not engage the resident in a repeated intruder stress paradigm (designated as “stress-susceptible” individuals) (105). Chronic social stress (housing with aggressive conspecific) results in transient CRH mRNA increases that normalize over time, despite subsequent development of severe adrenal insufficiency (106). Other studies do not report changes in CRH following repeated defeat stress (although samples were not stratified with respect to submissive behaviors) (107). However, social stress-induced increases in AVP mRNA expression have been reported by multiple groups (107–109), once again suggesting that prolonged activation of the HPA axis during social stress may be driven by this important ACTH co-secretagogue.

Unfortunately, the data on PVN function following social stress are largely limited to gene expression, limiting appreciation of cellular and neuroplastic adaptations that may be important to understanding mechanisms underlying social stress and its related pathologies. Moreover, these studies do not explore possible dynamic changes in PVN glucocorticoid signaling that are specific to social stress. These interesting discrepancies between PVN responses to environmentally imposed and social stress require additional attention, as they may inform essential differences between diseases linked to HPA axis hyper- vs. hypo-responsiveness (e.g., depression vs. PTSD/chronic fatigue).

## Summary

The weight of evidence overwhelmingly supports a dynamic neuroplasticity in the PVN as a result of chronic stress. Chronic stress has a demonstrable impact on cellular biosynthetic activity (cell size, neuropeptide expression); cell physiology (reduced inhibition and enhanced excitation); neurotransmission (receptor expression, innervation patterns); glucocorticoid feedback (rapid and genomic, local and upstream); and storage and release of ACTH secretagogues in the median eminence. Nearly all of these changes are predictive of enhanced potential for CRH and co-secretagogue release, which would be instrumental in driving HPA axis activity. Many of the most dramatic effects are related to upregulation of AVP signaling capacity in CRH neurons and at the pituitary, and underscore the potential importance of this co-localized and co-released peptide as an amplifier of the CRH signal normally initiated by these neurons. The mechanisms in place for promoting CRH neuronal activity occur in the context of a heightened glucocorticoid signal, indicating that neural drive is sufficient to largely overcome feedback inhibition under conditions of chronic stress. All of these findings highlight the importance of maintaining HPA axis stress reactivity even in the context of chronic stress, thereby defending the organism from real or perceived adversity.

Enhancement of the “potential energy” of the HPA axis is likely adaptive within the context of a period of prolonged, but manageable adversity. Indeed, upregulation of the PVN may be thought of as a component of “allostatic load,” an “adaptation through change” that keeps the system on line in the event of need (110). However, conditions of prolonged drive may have the capacity to push the system beyond the point of “adaptative” value and contribute to psychological and physiological pathologies associated with chronic stress. Likewise, prolonged engagement of PVN drive in the absence of an appropriate context would be sufficient to promote pathological features of HPA axis activation under inappropriate (e.g., non-stressed) conditions, as observed in diseases linked to stress (e.g., enhanced CRH and AVP mRNA expression and impaired dexamethasone suppression in depression).

Overall, as the “final common pathway” for stress integration by the brain, the PVN is ultimately responsible for both normal and pathological features of HPA axis stress responses. Despite progress to date, mechanisms underlying chronic stress-related PVN drive, modification of PVN secretagogue signaling, and synaptic plasticity are ill-defined. The significance of sex differences in PVN function is virtually unexplored. Failure to address these key issues in what is arguably the simplest component of stress reactivity imperils our ability to develop strategies to mitigate stress and diseases of stress adaptation.

## Author Contributions

Dr. JH wrote the article, along with intellectual and editorial contributions from Dr. JT.

## Conflict of Interest Statement

The authors declare that the research was conducted in the absence of any commercial or financial relationships that could be construed as a potential conflict of interest.
